# Spherical α-MnO_2_ Supported on N-KB as Efficient Electrocatalyst for Oxygen Reduction in Al–Air Battery

**DOI:** 10.3390/ma11040601

**Published:** 2018-04-13

**Authors:** Kui Chen, Mei Wang, Guangli Li, Quanguo He, Jun Liu, Fuzhi Li

**Affiliations:** Hunan Key Laboratory of Biomedical Nanomaterials and Devices, College of Life Sciences and Chemistry, Hunan University of Technology, Zhuzhou 412007, China; chenkui201802@163.com (K.C.); wangm20140900@126.com (M.W.); guangli010@163.com (G.L.); hequanguo@126.com (Q.H.)

**Keywords:** manganese dioxide, electrocatalyst, oxygen reduction reaction, Al-air battery, impedance, methanol tolerance

## Abstract

Traditional noble metal platinum (Pt) is regarded as a bifunctional oxygen catalyst due to its highly catalytic efficiency, but its commercial availability and application is often restricted by high cost. Herein, a cheap and effective catalyst mixed with α-MnO_2_ and nitrogen-doped Ketjenblack (N-KB) (denoted as MnO_2_-SM150-0.5) is examined as a potential electrocatalyst in oxygen reduction reactions (ORR) and oxygen evolution reactions (OER). This α-MnO_2_ is prepared by redox reaction between K_2_S_2_O_8_ and MnSO_4_ in acid conditions with a facile hydrothermal process (named the SM method). As a result, MnO_2_-SM150-0.5 exhibits a good catalytic performance for ORR in alkaline solution, and this result is comparable to a Pt/C catalyst. Moreover, this catalyst also shows superior durability and methanol tolerance compared with a Pt/C catalyst. It also displays a discharge voltage (~1.28 V) at a discharge density of 50 mA cm^−2^ in homemade Al–air batteries that is higher than commercial 20% Pt/C (~1.19 V). The superior electrocatalytic performance of MnO_2_-SM150-0.5 could be attributed to its higher Mn^3+^/Mn^4+^ ratio and the synergistic effect between MnO_2_ and the nitrogen-doped KB. This study provides a novel strategy for the preparation of an MnO_2_-based composite electrocatalyst.

## 1. Introduction

The efficiency of oxygen reduction reactions (ORR) and oxygen evolution reactions (OER) are critical to the energy conversion efficiency of metal air batteries because of their sluggish reaction kinetics [1]. In recent years, Pt-based materials have been developed as typical catalysts for ORR/OER with high catalytic activity [2,3,4]. However, their widespread application in commerce is seriously hindered, because the noble metal Pt is very expensive, and its reserves in earth are scarce. Therefore, in recent years, non-precious metal-based materials were extensively studied for developing a comparable candidate for Pt-based catalysts. Especially, a manganese-based material (such as manganese oxide) with satisfying catalytic activity has been developed recently, because it possesses a lot of advantages, including cheapness, abundance, environmental friendliness, structural flexibility, and bifunctional catalytic activity for ORR/OER [5,6,7,8,9]. Nevertheless, many factors have been found to play important roles in improving the catalytic activity of manganese dioxide for ORR. Firstly, the crystalline phase of manganese dioxide is critical. It has been reported that α-MnO_2_ exhibits better catalytic activity than other crystalline phases, because of its abundant di-μ-oxo bridges [10,11,12]. Secondly, micromorphology also has a great influence on its performance. Previous studies had shown that metal oxides with nanostructures exhibited good electrocatalytic performance because of their relatively large surface area and big pore volume, which exposes more active sites and facilitates full contact with electrolyte [13,14,15,16]. Thirdly, Mn^3+^ is believed to favor ORR/OER due to the single electron occupation in σ*-orbital (e_g_). Therefore, more content of Mn^3+^ in MnO_2_ could promote its electrocatalytic performance [17,18].

Although manganese-based materials have good electrocatalytic performance as reported, the superiority of synergic catalysts cannot be neglected. Recently, the research emphasis of manganese oxide has focused on ion doping and its composition with other materials, and results indicate that it has better catalytic property than bare manganese oxide. For example, Fe (or Co) ion-doped MnO_2_ nanosheets (MONSs) grown on the internal surface of macroporous carbon showed improved ORR catalytic activity compared with the un-doped one, because of the co-electrocatalytic function of MnO_2_ and Fe (or Co ion) [19]. Moreover, the catalyst of Mn_2_O_3_-doped MnO supported by reduced grapheme oxide (rGO) was proved to have a better ORR catalytic performance and stronger stability than that of pure MnO. It was believed that the coexisted metal oxide with different valences and rGO had promoted the catalytic performance [20]. In addition, carbon is one of the most important materials for electron transfer, and it could improve the catalytic activity. The coupling of MnO_2_ with carbon materials may improve its catalytic activities.

Herein, MnO_2_ spheres are synthesized by the redox reactions between K_2_S_2_O_8_ and MnSO_4_ in acid conditions (denoted as the SM method). These MnO_2_ spheres mixed with nitrogen-doped Ketjenblack (N-KB) are used as a catalyst for ORR/OER application. This study examines the morphology, structure, and electrochemical properties of MnO_2_ samples by scanning electron microscope (SEM), X-ray diffraction (XRD), X-ray photoelectron spectroscopy (XPS), and electrochemical testing. The relationships between the synthesized conditions and electrocatalytic activity of these MnO_2_-N-KB catalysts are also discussed. Finally, the proposed enhanced catalytic mechanism of these composited catalysts is investigated by controlling the Mn^3+^ content in MnO_2_. This work offers a new strategy for the scalable preparation of more efficient MnO_2_ bifunctional oxygen catalysts for ORR and OER.

## 2. Experiment

### 2.1. Preparation of MnO_2_

First, 1.69 g of manganese sulfate monohydrate, 1.74 g of potassium sulfate, and 2.71 g of potassium persulfate were dissolved into 80 mL of deionized water, agitating at least 5 min to form a homogenous aqueous solution. Then, 4 mL of 37% hydrochloric acid was added into the aqueous solution. After agitating another 5 min, the resulting solution was transferred into a 100-mL Teflon lined stainless steel shell autoclave, heated to 120 °C in an oven, and kept at the temperature for 12 h. After it was cooled to room temperature naturally, the as-obtained product was filtrated under vacuum with a membrane of 0.15-μm pore diameter and dried overnight at 80 °C. The obtained product was denoted as MnO_2_-SM120-12 (S: potassium persulfate; M: manganese sulfate).

For comparison, the control samples, such as MnO_2_-SM150-0.5 and MnO_2_-SM120-0.5, were also prepared. For the samples of MnO_2_-SM150-0.5, the synthesis process was the same with that of the MnO_2_-SM120-12, but the reaction temperature and reaction time were 150 °C and 30 min, respectively. For the samples of MnO_2_-SM120-0.5, the synthesis process was the same with that of the MnO_2_-SM120-12, but the reaction temperature and reaction time were 120 °C and 30 min, respectively.

### 2.2. Preparation of N-KB

First, 0.2 g of ketjenblack (KB) and 1.2 g of melamin were dispersed in 80 mL of deionized water by ultrasonic treatment for 30 min. Then, the resulting solution was enclosed into a 100-mL Teflon lined stainless steel shell autoclave, heated to 120 °C in an oven, and kept at the temperature for 24 h. After it was cooled down to room temperature naturally, the as-obtained product was filtrated under vacuum with a membrane of 0.15-μm pore diameter, and dried overnight at 80 °C. After being ground by agate mortar for more than 10 min, the resulting powder was transferred to a piece of porcelain boat, which was then covered with another piece of porcelain boat, and further wrapped by copper foil. The treated porcelain boat was placed into a tube furnace, and then heated to 650 °C for 2 h at a heating rate of 5 °C min^−1^ in argon flow. After that, it was naturally cooled down to room temperature, and the as-prepared sample was denoted as N-KB.

### 2.3. Characterization

The morphologies of the as-prepared catalysts were characterized by using the scanning electron microscope (FIB 600i, FEI, Hillsboro, OR, USA). The structures of these samples were carried out by X-ray diffraction (XRD, Rigaku D/Max 2550, Tokyo, Japan) with Cu-Kα radiation (λ = 1.5406 Å). The elemental and valence state analysis were characterized by X-ray photoelectron spectroscopy (XPS, K-Alpha1063 spectrometer, Thermo Scientific Co., Waltham, MA, USA).

### 2.4. Electrochemical Measurements

For electrochemical measurements, 2 mg of as-prepared MnO_2_ and 4 mg of N-KB were dispersed in 950 μL of anhydrous ethanol by sonication for 20 min. Then, 50 μL of Nafion solution (5 wt %) was added and sonicated for another 20 min to get a homogeneous catalytic ink. Then, 8 μL of the ink was loaded onto the surface of glassy carbon disk (the diameter is 5 mm, homemade electrode), and the catalyst loading amount was 0.0815 mg cm^−2^ (calculated by the mass of MnO_2_). For comparison, the commercial 20 wt % Pt/C (Johnson Matthey, Royston, UK) was also prepared with the same method.

Linear sweep voltammetry (LSV) and cyclic voltammetry (CV) ORR were performed using a RDE (rotating disk electrode) as working electrode, a double fluid boundary Ag/AgCl electrode as the reference electrode, and a platinum wire as the counter electrode in 0.1-M of KOH solution saturated with oxygen on a CHI760E electrochemical workstation. All of the potentials were finally converted to the values versus reversible hydrogen electrode. The ORR catalytic stabilities were evaluated by half-wave potential decay (Δ*E*_1/2_) before and after the accelerated durability test (ADT). The ADT was performed by using these catalysts in ORR for 5000 cycles. These experiments were carried out in O_2_-saturated 0.1 M of KOH solution at room temperature, and the voltage was selected from 0.57 V to 0.82 V (versus (Reversible Hydrogen Electrode) RHE) with a scan rate of 100 mV s^−1^. Methanol tolerance testing of catalysts was carried out in O_2_-saturated 0.1 M of KOH and 1.0 M of CH_3_OH-mixed electrolyte [21].

To further verify the ORR mechanism, the RRDE (rotating ring disk electrode) technique was used, and the peroxide percentage and the electron transfer number were calculated based on the equations, which were given as follows [22]:

(1) $${\mathrm{HO}}_{2}^{-}(\%)=200\times \frac{Ir/N}{Id+Ir/N}$$

(2) $$n=4\times \frac{Id}{Id+Ir/N}$$

where *I_d_* represents the disk current, *I_r_* represents the ring current, *N* represents the current collection efficiency of the Pt ring (0.37), and *n* means the electron transfer number [22,23].

The OER activities of the as-prepared samples were also carried out by RDE experiments at a scan rate of 10 mV s^−1^ with a rotation speed of 1600 rpm. The current density was operated from 0 to 16.5 mA cm^−2^. The long-term durability OER measurements of the catalysts were performed by using chronopotentiometry. The tests were conducted at a current density of 10 mA cm^−2^, and the test time was 8000 s. Finally, the EIS testes were scanned in the frequency range of 10^5^–0.1 Hz at 1.665 V (versus RHE) with the amplitude of 5 mV in 0.1 M of KOH solution [24].

### 2.5. Electrochemical Test of Al–Air Batteries

For an Al–air full batteries test, a polished aluminum stripe was used as anode, and 6 mol L^−1^ of a KOH solution containing 0.01 mol L^−1^ of Na_2_SnO_3_, 0.0075 mol L^−1^ of ZnO, and 0.0005 mol L^−1^ of In(OH)_3_ was used as electrolyte. The air electrodes were composed of a gas diffusion layer, a foam nickel current collector, and a catalytic layer. The catalytic layer was fabricated as follows. The as-prepared catalysts (10 mg), N-KB (30 mg), and the 60 wt % polytetrafluoroethylene (PTFE) aqueous solution (~50 mg) were mixed and agitated continuously until a paste appeared. Then, this paste was rolled with a glass rod until it turned into a 2 cm × 2 cm film. In the end, the film and the gas diffusion layer were pressed onto the two sides of nickel foam under the pressure of 10 MPa, and dried at 60 °C overnight. For comparison, the air electrode using commercial 20 wt % Pt/C catalyst was also fabricated with the same method. The full battery performance was measured with a Neware Battery Testing System (Shenzhen, China). A homemade electrochemical cell was used for Al–air battery measurements, with the net volume size of 50 mm × 32 mm × 50 mm, and an air hole with a diameter of 10 mm was used for the test [22,25].

## 3. Results and Discussion

### 3.1. Micromorphological and Microstructural Properties

**Figure 1:**
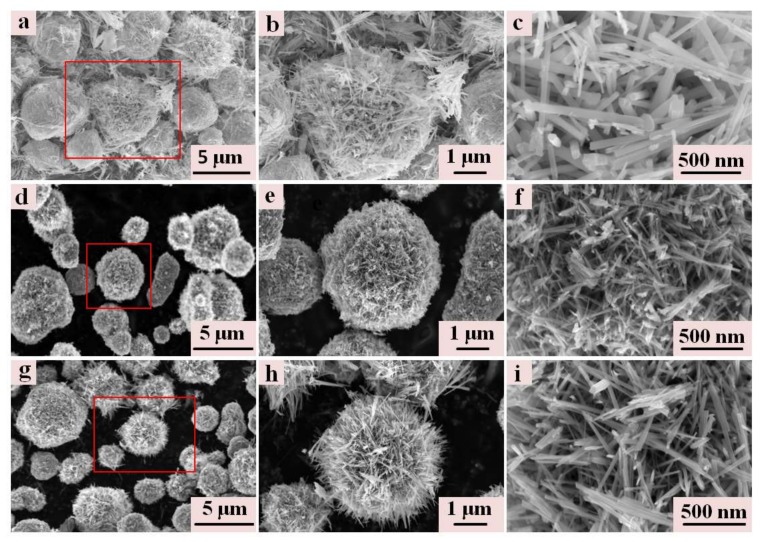
The SEM images of MnO_2_-SM120-12 (**a**–**c**); MnO_2_-SM120-0.5 (**d**–**f**); and MnO_2_-SM150-0.5 (**g**–**i**).

The micromorphology of the synthesized samples was characterized by SEM. Figure 1 shows the SEM images of MnO_2_-SM120-12 (a–c), MnO_2_-SM120-0.5 (d–f), and MnO_2_-SM150-0.5 (g–i). As we can see from these images, all three samples display spherical morphology. The average size of the MnO_2_-SM120-12 sample was ~5.0 μm, and these spheres were composed of nanorods with average diameters of 50 nm. The average diameter of the MnO_2_-SM150-0.5 spherical particles was ~4.2 μm, and are composed of nanorods with an average diameter of 27 nm. The spherical MnO_2_-SM120-0.5 sample shows an average diameter of ~2.9 μm, and is composed with nanorods (whose average diameter is about 20 nm). The MnO_2_-SM150-0.5 shows a smaller size than MnO_2_-SM120-12, which is mainly because of the higher hydrothermal reaction temperature and shorter hydrothermal reaction time. Although the manganese dioxide formed at a higher reaction speed during the production process, the samples with a smaller particle size could be produced in less reaction time [10]. However, the MnO_2_-SM150-0.5 sample shows a larger size than MnO_2_-SM120-0.5; this should be attributed to the effect of the higher reacting temperature, which causes a quicker reaction speed, and the quicker speed leads to a bigger size in the same reaction time.

**Figure 2:**
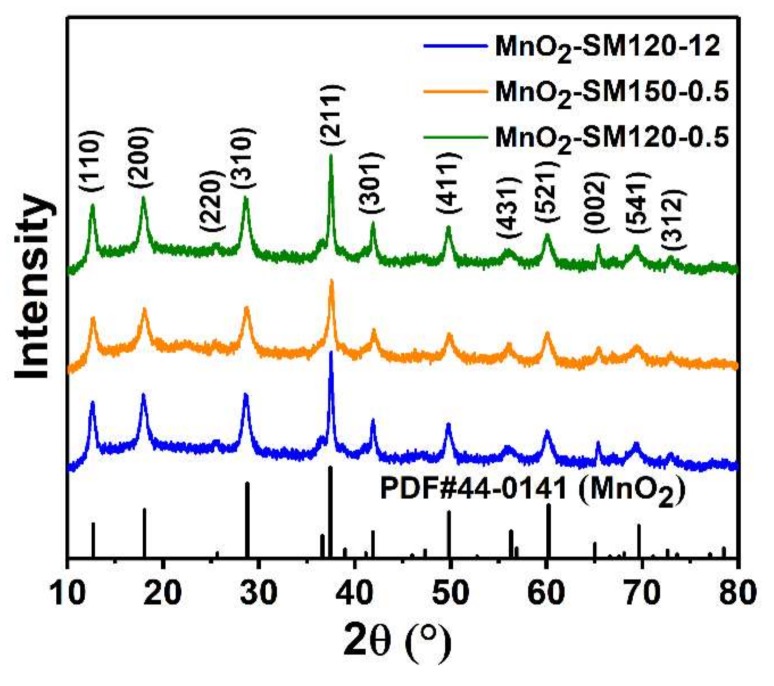
XRD patterns of MnO_2_-SM120-12 (green line), MnO_2_-SM120-0.5 (blue line), and MnO_2_-SM150-0.5 (orange line), the standard PDF card of MnO_2_ (PDF#44-0141) is carried out for comparison.

The structural characterization of MnO_2_-SM120-12 (green line), MnO_2_-SM120-0.5 (blue line), and MnO_2_-SM150-0.5 (orange line), were carried out and compared by XRD. As shown in these XRD patterns (Figure 2), the diffraction peaks located at the 12.8, 18.1, 25.8, 28.8, 37.5, 42.0, 49.9, 56.9, 60.3, 65.1, 69.7 and 72.7 positions in all three samples belonged to the (110), (200), (220), (310), (211), (301), (411), (431), (521), (002), (541) and (312) facets, respectively. These sharp peaks indicated the good crystallization property of the samples, and there no other diffraction peaks were observed in these patterns, further indicating the good crystallization property of these samples. The standard card of α-MnO_2_ (PDF#44-0141) was presented for comparison, and these diffraction peaks matched well with this standard card. It revealed that these as-synthesized MnO_2_ particles are α-MnO_2_ particles. Obviously, the diffraction peaks intensity of MnO_2_-SM150-0.5 (orange line) were inferior to those of the other two samples; this is probably because more defects existed in this sample, which could improve its catalytic activities. Moreover, it has been generally accepted that α-MnO_2_ exhibits better catalytic activity than other crystalline phases because of its abundant di-μ-oxo bridges [11]. Thus, the α-MnO_2_ particles prepared here could be used for high-performance catalytic application.

**Figure 3:**
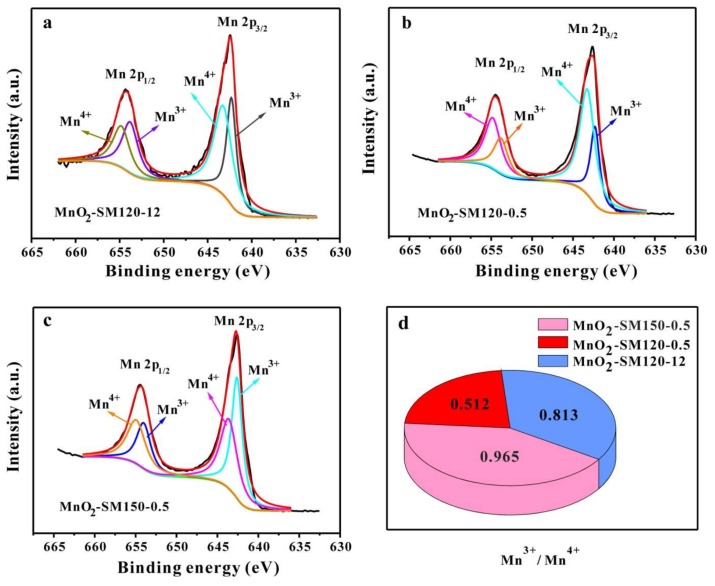
XPS spectra of Mn 2*p* for MnO_2_-SM120-12 (**a**); MnO_2_-SM120-0.5 (**b**); MnO_2_-SM150-0.5 (**c**); and the Mn^3+^/Mn^4+^ values of the three samples (**d**).

XPS spectra were carried out for further elemental and valence state analysis of MnO_2_-SM120-12, MnO_2_-SM120-0.5, and MnO_2_-SM150-0.5 samples. As shown in Figure 3, the high-resolution XPS spectra of Mn 2*p* for MnO_2_-SM120-12 (Figure 3a), MnO_2_-SM120-0.5 (Figure 3b), and MnO_2_-SM150-0.5 (Figure 3c) are presented, and four peaks located at 642.30, 643.25, 653.80, and 654.80 eV were obtained by peak-differentiating technique. These peaks were assigned to the Mn^3+^ (2*p*_3/2_), Mn^4+^ (2*p*_3/2_), Mn^3+^ (2*p*_1/2_), and Mn^4+^ (2*p*_1/2_) species, respectively [6,26,27]. Moreover, based on the XPS results, the perk areas of Mn^3+^ and Mn^4+^ in each sample are presented in Table 1, and the ratio values of Mn^3+^/Mn^4+^ (Figure 3d) for MnO_2_-SM120-12, MnO_2_-SM120-0.5, and MnO_2_-SM150-0.5 were calculated as 0.813, 0.512 and 0.965, respectively. As shown in Figure 3d, obviously, the Mn^3+^/Mn^4+^ ratio in MnO_2_-SM150-0.5 (0.965) was higher than that in MnO_2_-SM120-12 (0.813), which should be ascribed to the higher reacting temperature that causes the faster reaction rate. It is believed that the faster reaction rate could cause more Mn^3+^ content in MnO_2_. A higher content of Mn^3+^ in MnO_2_ can lead to a better electrocatalytic performance, due to the single electron occupation in the σ*-orbital (e_g_) of Mn^3+^ [17,18,28]. In addition, a shorter reacting time could produce MnO_2_-SM150-0.5 with a smaller size (Figure 1), which would impact its electrocatalytic performance. The Mn^3+^/Mn^4+^ ratio in MnO_2_-SM150-0.5 (0.965) was also higher than that in MnO_2_-SM120-0.5 (0.512), which should be because the faster reaction rate could cause more Mn^3+^ content in MnO_2_ [10].

### 3.2. ORR Activity and Stability

**Figure 4:**
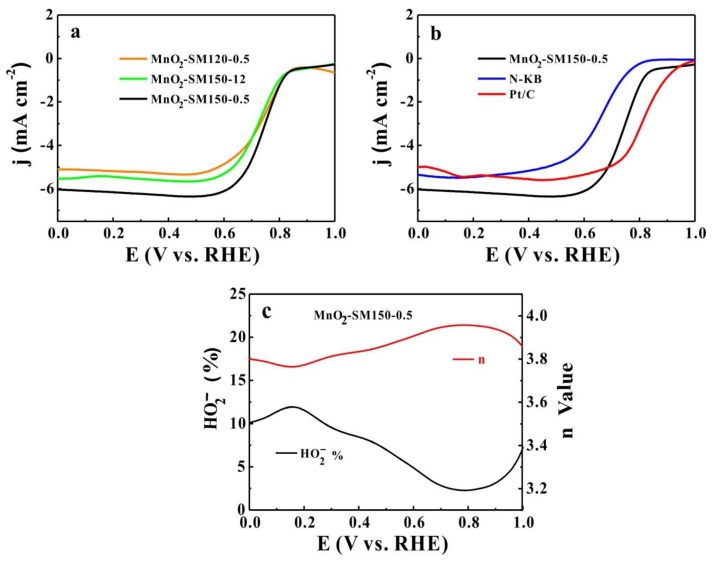
(**a**) Oxygen reduction reaction (ORR) linear sweep voltammetries (LSVs) of MnO_2_-SM120-12, MnO_2_-SM120-0.5, and MnO_2_-SM150-0.5 in 0.1 M of KOH solution at a scan rate of 10 mV s^−1^ with a rotation speed of 1600 rpm; (**b**) ORR LSVs of MnO_2_-SM120-0.5, N-KB, and 20% JM. Platinum (Pt)/C in 0.1 M of KOH solution at a scan rate of 10 mV s^−1^ with a rotation speed of 1600 rpm; (**c**) Percentage of peroxide (${\mathrm{HO}}_{2}^{-}\%$) and the electron transfer number (*n*) of MnO_2_-SM150-0.5.

The LSV curves for the ORR of MnO_2_-SM120-12, MnO_2_-SM120-0.5, and MnO_2_-SM150-0.5 are shown in Figure 4a; these measurements were carried out in 0.1 M of KOH solution at a rotating speed of 1600 rpm. The MnO_2_-SM150-0.5 sample showed a better ORR catalytic performance than MnO_2_-SM120-12 at the same test conditions, because the size of the particles in the MnO_2_-SM150-0.5 sample was smaller, and it contained more Mn^3+^ in comparison with the MnO_2_-SM120-12 sample [28,29]. It is clear that MnO_2_-SM150-0.5 also exhibited a better ORR catalytic performance than the MnO_2_-SM120-0.5 samples, mainly because of the higher Mn^3+^ content. As shown in Figure 4b, MnO_2_-SM150-0.5 (supported on N-KB) exhibited a much better ORR catalytic performance than bare N-KB, with a half-wave potential of 0.76 V and a limiting current density of 6.0 mA cm^−2^. This phenomenon should be attributed to the synergetic catalytic activity of α-MnO_2_ and N-KB. It is believed that the intrinsically abundant di-μ-oxo bridges in α-MnO_2_ could facilitate the ORR process [7,12,19,28]. As observed, the limiting current density (6.0 mA cm^−2^) of MnO_2_-SM150-0.5 (supported on N-KB) was higher than Pt/C (~5.0 mA cm^−2^), despite its lower half-wave potential (0.76 V) compared with Pt/C (~8.2 V). As shown in Figure 4c, the average *n* value of MnO_2_-SM150-0.5 was 3.85 (from 3.78 to 3.95), confirming a four-electron (4e^−^) oxygen reduction mechanism.

**Figure 5:**
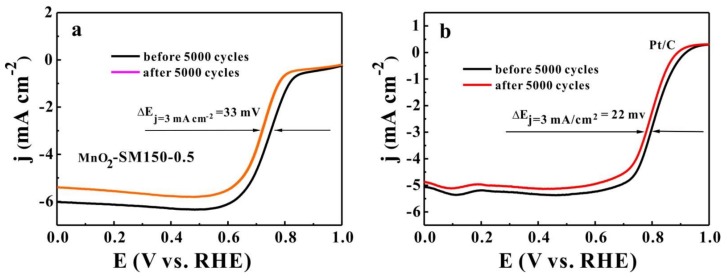
LSV curves of MnO_2_-SM150-0.5 (**a**) and Pt/C (**b**) before and after the accelerated durability test (ADT). The ADT was performed by subjecting the catalyst to 5000 circles from 0.57 V to 0.82 V (vs. RHE) in an O_2_-saturated 0.1 M of KOH solution at room temperature at a scan rate of 100 mV s^−1^.

The catalytic stabilities of the MnO_2_-SM150-0.5 and Pt/C samples in ORR were evaluated by the half-wave potential decay (Δ*E*_1/2_) before and after the accelerated durability test (ADT) [30]. The ADT was performed by using these catalyst in an ORR for 5000 cycles. These experiments were carried out in an O_2_-saturated 0.1 M of KOH solution at room temperature, and the voltage was selected from 0.57 V to 0.82 V (versus RHE) with a scan rate of 100 mV s^−1^. As shown in Figure 5a, the half-wave potential of the MnO_2_-SM150-0.5 sample (supported on N-KB) exhibited a negative shift of ~33 mV after 5000 cycles; this result is slightly higher than that of Pt/C (~22 mV, Figure 5b), which is probably because of the inferior electroconductibility of MnO_2_ compared with the noble metal Pt.

### 3.3. OER Activity and Stability

**Figure 6:**
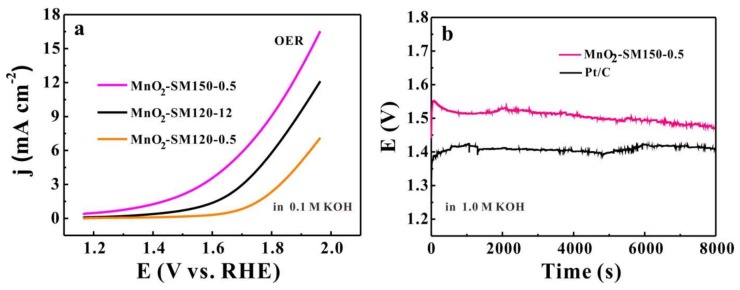
The oxygen evolution reaction (OER) performances of MnO_2_-150-0.5 and MnO_2_-120/12 are evaluated by LSVs in 0.1 M of KOH solution at a scan rate of 10 mV s^−1^ with a rotation speed of 1600 rpm (**a**); the stability test of OER activity of MnO_2_-SM150-0.5 and Pt/C samples in 1.0 M of KOH solution at a current density of 10 mA cm^−2^ for 8000 s (**b**).

In addition, the OER activities of three as-prepared samples (MnO_2_-SM120-12, MnO_2_-SM120-0.5, and MnO_2_-SM150-0.5) were carried out for further comparing their electrocatalytic performances, and they were tested by RDE experiments at the scan rate of 10 mV s^−1^ with a rotation speed of 1600 rpm. Generally, OER activities are judged by the potential at the current density from 0 mA cm^−2^ to 16.5 mA cm^−2^ [31]. As presented in Figure 6a, MnO_2_-150-0.5 showed a more negative shift than the MnO_2_-120-12 and MnO_2_-SM120-0.5 samples when the current density increased from 0 mA cm^−2^ to 16.5 mA cm^−2^, which means that the MnO_2_-15-0.5 could catalyze OER at a lower overpotential than the MnO_2_-120-12 and MnO_2_-SM120-0.5 samples. In other words, the MnO_2_-150-0.5 sample exhibited much better OER kinetic behavior than the MnO_2_-120-12 and MnO_2_-SM120-0.5 samples. Similar to the ORR, the higher content of Mn^3+^ and smaller size of the α-MnO_2_ nanorods also played a vital role on the OER. Moreover, the stability tests of the OER activity of the MnO_2_-SM150-0.5 and Pt/C samples are presented. The long-term durability measurements of the catalysts were performed by using chronopotentiometry. The tests were conducted at a current density of 10 mA cm^−2^, and the test time was 8000 s. As presented in Figure 6b, after reaction for 8000 s, MnO_2_-SM150-0.5 showed a good stability in OER, but slightly not as good as Pt/C, indicating that the practical catalytic performance of MnO_2_-SM150-0.5 needs further improvement to replace the Pt/C.

### 3.4. EIS Performance

**Figure 7:**
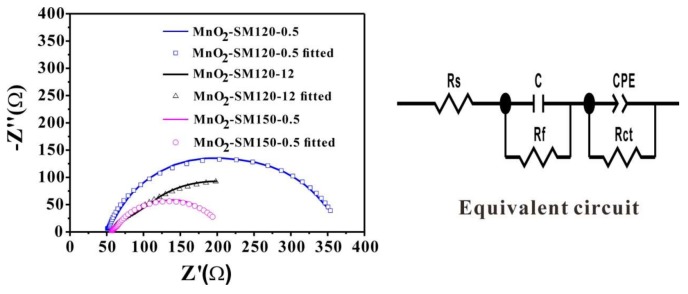
Nyquist plots of MnO_2_-120-0.5, MnO_2_-120-12, and MnO_2_-150-0.5 samples obtained from electrochemical impedance spectroscopy (EIS) measurements in 0.1 M of KOH solution at 1.665 V (vs. RHE) and the inserted is the corresponding equivalent circuit.

The charge transfer efficiency of the catalyst plays an important role in the OER process, as the high electron transfer efficiency could indicate the high catalytic activity. The electrochemical impedance spectroscopy (EIS) is a good method for understanding charge transfer efficiency, because the arc radius size of the EIS curve could indicate the value of electrical resistance. The lower resistance of the sample implies the high electroconductibility. Thus, the EIS method is used for deep insights into the OER process. The EIS tests were scanned in the frequency range of 10^5^–0.1 Hz at 1.665 V (versus RHE) with the amplitude of 5 mV in 0.1 M of KOH solution [17,32]. The Nyquist plots are shown in Figure 7, in which the EIS data (Figure 7 left) have been fitted according to the equivalent circuit (Figure 7 right). The equivalent circuit consisted of R_s_, R_f_, R_ct_, C, and CPE, representing the uncompensated solution resistance, intrinsic resistance of the catalyst, charge transfer resistance, capacitance of catalyts, and constant phase element of the double layer, respectively. All of the fitting parameters are listed in Table 2. The R_s_ relating to the uncompensated solution of the MnO_2_-120-0.5, MnO_2_-120-12, and MnO_2_-150-0.5 samples are 55.72 Ω, 61.75 Ω, and 62.01 Ω, respectively. The R_ct_ relates to the reaction kinetics; MnO_2_-150-0.5 exhibited a lower charge transfer resistance (146.4 Ω) than those of the MnO_2_-120-12 (257.1 Ω) and MnO_2_-120-0.5 (313.5 Ω) samples. Note that it is in good accordance with the OER performance.

### 3.5. Methanol Tolerance Performance

**Figure 8:**
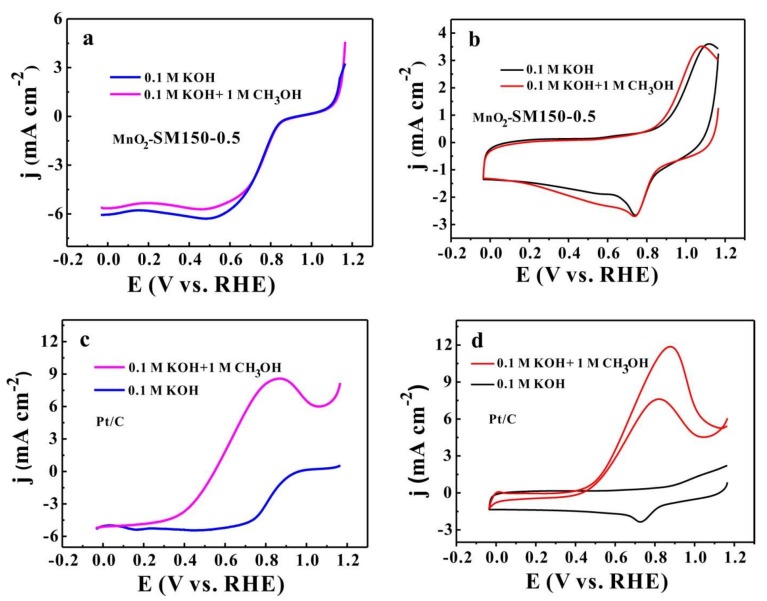
LSV curves of MnO_2_-SM150-0.5 (**a**) and Pt/C (**c**) in an O_2_-saturated 0.1 M of KOH electrolyte with (purple line) and without (blue line) 1.0 M methanol; cyclic voltammetry (CV) curves of MnO_2_-SM150-0.5 (**b**) and Pt/C (**d**) at 20 mV s^−1^ in an O_2_-saturated 0.1 M of KOH electrolyte with (red line) and without (black line) 1.0 M methanol.

The methanol tolerance of the catalysts is usually used to evaluate the performance of ORR catalysts in DMFCs (direct methanol fuel cells). The LSV and CV experimental groups (by using the MnO_2_-SM150-0.5 sample (supported on N-KB) and Pt/C samples as catalysts) and control group were carried out in an O_2_-saturated 0.1 M of KOH electrolyte for testing the methanol tolerance. These results are presented in Figure 8. As we can see in Figure 8a, when the ORR process was carried out in an O_2_-saturated 0.1 M of KOH electrolyte with 1.0 M of methanol, the MnO_2_-SM150-0.5 catalyst exhibited excellent methanol tolerance properties, because there was no negative shift of onset potential and no oxidation currents of methanol, but rather only a slight decrease of the limiting current density (~0.25 mA cm^−2^) (Figure 8b). However, a strong oxidation current of methanol is shown in Figure 8d with the Pt/C sample by comparison with the background line. Moreover, a larger negative shift of onset potential for ORR (from ~1.0 V to ~0.52 V) is observed in Figure 8c, indicating the poor methanol tolerance of the Pt/C sample in comparison with the MnO_2_-SM150-0.5 catalyst [9].

### 3.6. Application in Al-Air Battery

**Figure 9:**
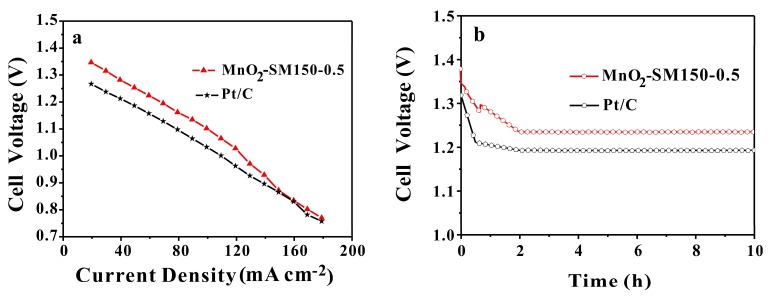
(**a**) Polarization curves of Al–air batteries and (**b**) discharge curves at a constant current density of 50 mA cm^−2^.

For further evaluating the practical catalytic performance of the MnO_2_-SM150-0.5 sample in an Al–air battery, cell voltages at various current densities and constant current discharge tests were carried out. The commercial Pt/C sample was also investigated for comparison. As the results show in Figure 9a, overall, the cell polarization curve with the MnO_2_-SM150-0.5 sample was better than that of Pt/C. Specifically, when the discharge current density was lower than 150 mA cm^−2^, the cell voltages of MnO_2_-SM150-0.5 were higher than those of the Pt/C sample. However, the cell voltages of the MnO_2_-SM150-0.5 sample were almost equal to those of Pt/C at the range of 150 mA cm^−2^–180 mA cm^−2^. As shown in Figure 9b, MnO_2_-SM150-0.5 showed a discharge voltage platform of ~1.24 V, which was slightly higher than that of Pt/C (~1.19 V) at the end of the discharge test with a constant current density of 50 mA cm^−2^ in homemade Al–air batteries. However, it can be observed that the MnO_2_-SM150-0.5 sample took about 2 h to achieve the smoothing discharge voltage platform, which was longer than that of Pt/C (less than 1 h), indicating that the practical catalytic performance of MnO_2_-SM150-0.5 needs further improvement to replace the Pt/C.

## 4. Conclusions

In this work, three kinds of α-MnO_2_ microspheres composed with nanorods were synthesized in acid conditions using K_2_S_2_O_8_ and MnSO_4_ as raw materials by a facile hydrothermal process. The influences of Mn^3+^ content on the electrocatalytic activity of ORR/OER were also studied. These results demonstrated that catalysts with more Mn^3+^ content play an important role in electrocatalytic application. Especially, the MnO_2_-SM150-0.5 sample with higher Mn^3+^ content showed a better electrocatalytic performance than the MnO_2_-SM120-0.5 and MnO_2_-SM120-12 samples. The half-wave potential (*E*_1/2_) of the MnO_2_-SM150-0.5/N-KB sample was 0.76 V (versus RHE), and the limiting current density was about 6.0 mA cm^−2^. This result could be comparable to those of Pt/C (0.82 V and ~5.0 mA cm^−2^, respectively). Moreover, the MnO_2_-SM150-0.5 sample showed an excellent methanol tolerance compared to the Pt/C sample. In addition, the MnO_2_-SM150-0.5 sample exhibited good ORR catalytic stability; as its half-wave potential only negatively shifted ~33 mV after 5000 cycles. Besides, the MnO_2_-SM150-0.5 sample exhibited a higher discharge voltage (1.28 V) at a density of 50 mA cm^−2^ than the Pt/C catalyst (1.19 V) when used in homemade Al–air batteries as cathode catalysts. Thus, this strategy for the preparation of α-MnO_2_ could provide a scalable preparation method for significant ORR/OER application.

## Figures and Tables

**Table 1:** The XPS dates of Mn 2*p* for MnO_2_-SM120-12; MnO_2_-SM120-0.5; MnO_2_-SM150-0.5; and the corresponding perk areas and Mn^3+^/Mn^4+^ values.

Samples	Species	Peak Position (eV)	Peak Area	Mn^3+^/Mn^4+^
MnO_2_-SM150-0.5	2*p *_3/2_ Mn^3+^	642.60	36429.72	0.965
	2*p*_3/2_ Mn^4+^	643.60	33877.38	
	2*p*_1/2_ Mn^3+^	654.00	18763.28	
	2*p*_1/2_ Mn^4+^	654.90	23322.43	
MnO_2_-SM120-0.5	2*p*_3/2_ Mn^3+^	642.30	20298.63	0.512
	2*p*_3/2_ Mn^4+^	643.25	44859.42	
	2*p*_1/2_ Mn^3+^	653.80	14298.36	
	2*p*_1/2_ Mn^4+^	654.80	22753.79	
MnO_2_-SM120-12	2*p*_3/2_ Mn^3+^	642.30	29778.53	0.813
	2*p*_3/2_ Mn^4+^	643.25	45449.07	
	2*p*_1/2_ Mn^3+^	653.80	23045.48	
	2*p*_1/2_ Mn^4+^	654.80	19499.66	

**Table 2:** Component values of fitted equivalent circuit based on the Nyquist plots.

Sample	R_s_ (Ω)	R_f_ (Ω)	R_ct_ (Ω)	C (F)	CPE-T	CPE-P
MnO_2_-SM150-0.5	55.72	4.77	146.4	0.001010	0.002368	0.842
MnO_2_-SM120-12	61.75	8.89	257.1	0.005600	0.003926	0.692
MnO_2_-SM120-0.5	62.01	9.69	313.5	0.000031	0.002031	0.895
